# The effect of extracellular potassium concentration on the oscillation frequency of the pacemaker nucleus in the weakly electric fish *Apteronotus leptorhynchus*

**DOI:** 10.1007/s00359-024-01719-0

**Published:** 2024-10-23

**Authors:** Masashi Kawasaki, Günther K. H. Zupanc

**Affiliations:** 1 Department of Biology, University of Virginia, Charlottesville, VA 22904 USA; 2 Laboratory of Neurobiology, Department of Biology, Northeastern University, Boston, MA 02115 USA

**Keywords:** Behavioral sexual dimorphism, *Apteronotus leptorhynchus*, Electric fish, Electric organ discharge, Pacemaker nucleus, Extracellular potassium concentration, Osmolarity

## Abstract

The weakly electric brown ghost knifefish (*Apteronotus leptorhynchus*) exhibits a pronounced sexual dimorphism in its electric behavior—males discharge at higher frequencies than females, with little overlap between the sexes. The frequency of these electric organ discharges is controlled by the frequency of the synchronized oscillations of the medullary pacemaker nucleus. Previous studies have suggested that sex-specific differences in the morphology and gene expression pattern of the astrocytic syncytium that envelopes the pacemaking neural network cause differences in its capacity to buffer the extracellular concentration of K^+^. This change in the K^+^ buffering capacity affects the K^+^ equilibrium potential of the neurons constituting the neural network, which in turn modulates the frequency of the pacemaker nucleus. In the present study, we have tested a critical element of this hypothesis by examining whether, and how, changes in the extracellular K^+^ concentration influence the frequency of the pacemaker nucleus oscillations. By using an in vitro preparation of the pacemaker nucleus, the results of this investigation demonstrate that exposure of this nucleus to acutely increased/decreased concentrations of K^+^ in the perfusate (while maintaining osmolarity) leads to concentration-dependent increases/decreases in the frequency of the synchronized oscillations generated by the pacemaker nucleus.

## Introduction

Sexual dimorphism in behavior is a well-characterized phenomenon in ethology, yet the cellular processes that produce such differences between males and females are less well understood. The weakly electric brown ghost knifefish (*Apteronotus leptorhynchus*) provides an excellent model system for studying these processes, as its electric organ discharge (EOD) can be readily monitored, is sexually dimorphic, and the neural substrate controlling the electric behavior is well characterized (for reviews see Zupanc 2017, 2020).

The electric organ of this gymnotiform fish is composed of immensely enlarged axonal terminals of modified spinal motoneurons, referred to as electromotoneurons. By means of this electric organ, both sexes generate continuous electric discharges at frequencies that are highly constant in a given individual but variable among individuals within the species-specific range of 650–1000 Hz (de Oliveira-Castro 1955; Bennett 1971; Waxman et al. 1972; for review see Zupanc and Bullock 2005). Males discharge at higher frequencies than females, with little overlap between the sexes (Meyer et al. 1987). The development and maintenance of this sexual dimorphism in the frequency of the electric organ discharge (*f*_EOD_) is under control of steroid hormones. Chronic administration of β-estradiol results in a pronounced frequency decrease (Meyer et al. 1987; Schaefer and Zakon 1996; Zupanc et al. 2014).

The EOD is driven, in a one-to-one fashion, by command spikes originating from the pacemaker nucleus (Pn) in the medulla oblongata. Thus, *f*_EOD_ equates the frequency of the synchronized oscillations of the pacemaker nucleus (*f*_Pn_) (for review see Dye and Meyer 1986). In midsized adult fish, this brainstem nucleus consists of approximately 200,000 cells, of which 5400 have been identified as neurons (Sîrbulescu et al. 2014). The neural network essential for producing the neural oscillations of the Pn is formed, on average, by 87 pacemaker cells and 20 relay cells. (The function of the third neuronal subtype—small interneurons—is unknown.) The output of the Pn is conveyed, via the axons of the relay cells, to the electromotoneurons in the spinal cord. Computational modeling has shown that the network composition of the Pn is optimized for generating synchronized sustained oscillations (Ilieş and Zupanc 2023).

The pacemaker and relay cells of the Pn are embedded in a dense meshwork of glial fibrillary acidic protein (GFAP)-immunopositive astrocytes that form a syncytium coupled by gap junctions expressing the transmembrane protein connexin-43. The number of astrocytes has been estimated to total approximately 40,000 (Sîrbulescu et al. 2014), thus outnumbering the pacemaker and relay cells roughly 400-fold. The morphology and expression pattern of these astrocytes and their junctions exhibit pronounced sexual dimorphisms (Zupanc et al. 2014). Both the surface area of pacemaker and relay cells covered by the astrocytes, as well as the overall intensity of GFAP expression within the Pn and the abundance of connexin-43 immunoreactivity in the vicinity of pacemaker cells, are significantly higher in females than in males. Like the decrease in *f*_EOD_, ‘female’-like astrocytic morphology and GFAP expression can be induced by chronic implantation of β-estradiol into males (Zupanc et al. 2014).

Since astrocytic syncytia play a major role in the buffering and redistribution of extracellular potassium during high-frequency firing in the brain firing (Lux et al. 1986; Somjen 2004), it has been suggested that the sex-specific differences in the morphology and gene expression pattern of the astrocytes and their junctions in the Pn lead to modulation of this function (Zupanc 2017, 2020). According to this hypothesis, the K^+^ buffering capacity is elevated in females, compared to males. This effect results in a decrease in the extracellular potassium concentration ([K^+^]_out_) and, thus, of the K^+^ equilibrium potential, which, in turn, leads to a decrease in *f*_Pn_. This notion is in agreement with simulations based on computational models of individual neurons and the whole neural network of the Pn (Zupanc et al. 2019; Hartman et al. 2021). The idea that the excitability of neurons and their firing pattern can be modulated through changes in [K^+^]_out_ receives further support from the results of mathematical modeling and physiological experiments obtained in several other neural systems (Alger and Teyler 1978; Poolos and Kocsis 1990; Brumberg et al. 2000; Contreras et al. 2021).

In the present study, we examined the effects of [K^+^]_out_ on *f*_Pn_ by systematically changing the extracellular K^+^ concentration in an in vitro preparation of the Pn and by assessing possible effects on this frequency through extracellular recording.

## Materials and methods

### Animals

*A. leptorhynchus* was obtained through a tropical fish importer (AliKhan Tropical Fish, Jamaica, New York). Several tens of fish were used for this study, including its exploratory phase. The data presented in this paper were collected through recordings of the neural activity of the Pn of 8 individuals that met the following data inclusion criteria: (1) recovery of pacemaking activities within 30 min of surgical excision of the Pn; (2) in vitro pacemaking frequency within 20% (mean 11.3%) of *f*_EOD_ of intact fish before the surgery; (3) full synchronization of the entire nucleus as confirmed by identical frequencies from multiple recording locations within the nucleus; (4) maintenance of conditions (1–3) for more than 1 h.

The size of the 8 fish that met the data-inclusion criteria ranged from 115–164 mm total length and 1.9–9.1 g body weight. Their *f*_EOD_ values, adjusted to a reference water temperature of 26.5 °C, ranged from 743–964 Hz. Gonadal inspection revealed eggs in 3 fish (with *f*_EOD_ values adjusted to 26.5 °C of 760, 762, and 778 Hz) and testes in 3 fish (with *f*_EOD_ values adjusted to 26.5 °C of 897, 914, and 964 Hz). In 2 fish (with *f*_EOD_ values adjusted to 26.5 °C of 743 and 893 Hz) no gonads could be identified. Gonadal inspection and/or the *f*_EOD_ values indicate that 4 of the fish were male, whereas the other 4 were female.

### Dissection of Pn

In vitro preparations of the Pn were made according to the previously published protocols (Meyer 1984; Dye 1988, 1991) with the following modifications. After measurement of the EOD frequency of the intact fish, it was submerged into 0.075% 2-phenoxyethanol in an ice water bath until opercular and fish movements ceased. The spinal cord and the cranial nerves were transected with a pair of curved scissors, and the brain was transferred to a Petri dish with cold (4 °C) artificial cerebrospinal fluid (ACSF; NaCl, 114 mM; NaH_2_PO_4_, 1.25 mM; MgSO_3_, 1.1 mM; CaCl_2_, 1.1 mM; NaHCO_3_, 25 mM; glucose, 10 mM). Following removal of meninges, the Pn and surrounding tissue were dissected out with a pair of iris scissors (Fine Science Tools, 15,000–08). The size of the dissected tissue block, including the Pn and surrounding tissue, was approximately 1.8 mm × 1.8 mm × 0.7 mm.

### Extracellular recordings

**Fig. 1:**
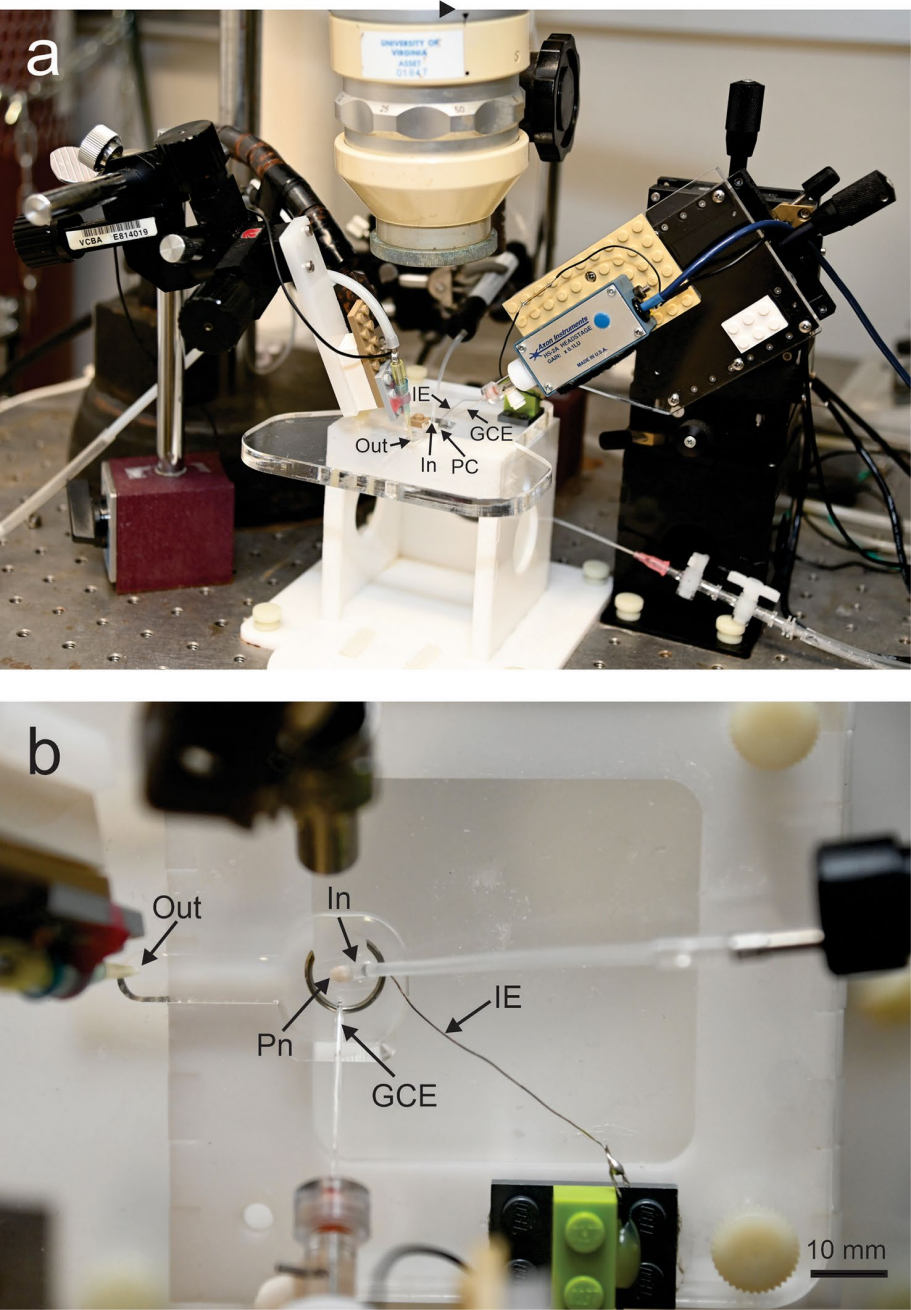
Experimental setup for extracellular and intracellular recordings. **a** Overview. **b** Magnified view from above. The isolated pacemaker nucleus (Pn) is placed, with its ventral side up, in the flat-bottomed perfusion chamber (PC). The low volume of this chamber (~ 400 µL), in combination with the high flow rate through the inlet (In) and outlet (Out) lines (50–100 µL/s), enabled effective and fast manipulation of the extracellular tissue environment. *GCE* glass capillary electrode, *IE* indifferent electrode

The tissue block with the Pn was transferred to a custom-made submerge-type in vitro recording chamber and anchored with a slice clamp (Warner Instruments, SHD-42/15), ventral side up (Fig. 1). The nucleus was supplied with ACSF, saturated with a mixture of 95% oxygen and 5% CO_2_ and kept at 26.5 °C with an inline temperature controller (Warner Instruments, Hamden, Connecticut; TC-324C). The bath volume was ~ 400 µL, and the rate of tissue perfusion with the ACSF was 50–100 µL/s.

Extracellular field potential was recorded with glass capillary electrodes (tip diameter ~ 30 µm) filled with ACSF and connected to a patch clamp amplifier (Axon Instruments, Foster City, California; AxoClamp-2B) set to the current clamp mode. The amplitude of the field potential from a fully synchronized nucleus was 0.3–1 mV. The potential was fed to a digitizer (National Instruments, Austin, Texas; PCI-6229) and subjected to online FFT processing by a custom-written MATLAB program (MathWorks, Natick, Massachusetts). The frequency values mentioned in this paper are the peak frequencies of the amplitude spectra. The time and frequency resolutions of the online amplitude spectrum were 2 s and 1 Hz, respectively.

### Temperature adjustment of EOD frequency

Since the *f*_EOD_ of *A. leptorhynchus* is temperature dependent (Enger and Szabo 1968; Zupanc et al. 2003), we adjusted all frequencies reported in this paper to a reference temperature of 26.5 °C so that discharge frequencies at different ambient temperatures could be compared. This adjustment of a frequency ${f}_{1}$ (in Hz) measured at a temperature ${T}_{1}$ (in °C) to the reference temperature ${T}_{2}=26.5$ °C was calculated as

$${f}_{2}={f}_{1} {{Q}_{10}}^{\frac{{T}_{2}-{T}_{1}}{10}}$$

where ${f}_{2}$ is the expected frequency at ${T}_{2}$ and ${Q}_{10}=1.56$, as determined empirically previously (Zupanc et al. 2003).

### Statistical analysis

Statistical analysis of the data was performed using the software package IBM SPSS Statistics Version 28.0.0.0 (IBM Corporation, Armonk, New York). Pearson Correlation Analysis was used to measure the strength of the linear association of shifts in *f*_Pn_ with different [K^+^]_out_. The significance level was set at *p* < 0.05 (2-tailed).

## Results

### Effects on Pn oscillation frequency of changes in osmolarity and sodium concentration of the perfusate

In a previous study, Dye (1991) examined the effect of an increase in [K^+^]_out_ from 3.25 mM to 20 mM on neural oscillations of the Pn. The increase in osmolarity by this addition of salt was compensated for by an equimolar reduction in the concentration of NaCl from 124 mM to 107.25 mM. However, since subsequently it was shown that sodium currents are involved in the regulation of the pacemaker rhythm (Smith and Zakon 2000), it is unclear whether the effects observed by Dye (1991) were caused by the increase in [K^+^]_out_ and/or by the reduction in the extracellular concentration of sodium ([Na^+^]_out_). Thus, in an initial experiment, we examined how the oscillation frequency of the Pn is affected by changes in [Na^+^]_out_ and/or osmolarity.

**Fig. 2:**
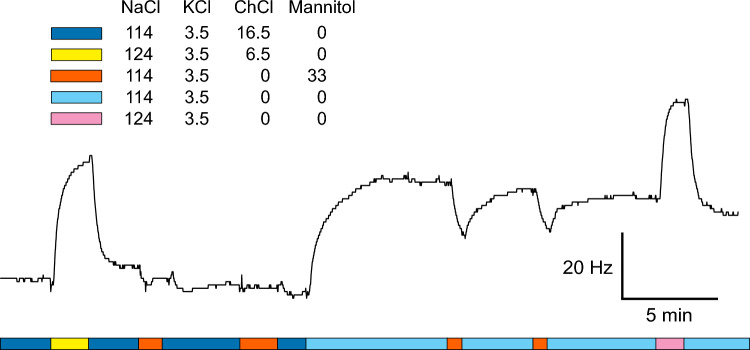
Effect of changes in osmolarity and/or sodium concentration in the perfusate on the oscillation frequency of the Pn. After the nucleus exhibited a stable oscillation frequency in the base solution containing 114 mM NaCl, and 16.5 mM choline chloride, the concentration of NaCl was increased to 124 mM, while the concentration of choline chloride was reduced to 6.5 mM to maintain osmolarity. The Pn responded to this change with a large increase in oscillation frequency. Replacing choline chloride in the base solution with an equimolar amount of mannitol did not have a notable effect. On the other hand, removal of mannitol from the solution led to a marked increase in oscillation frequency. Short pulses of solutions to which 33 mM mannitol or 16.5 mM choline chloride was added evoked drops in oscillation frequency. Stimulation of the nucleus with a solution containing an elevated concentration of NaCl (124 mM) but neither mannitol nor choline chloride for osmolarity compensation resulted in a significant frequency increase. The oscillation frequency at the beginning of the trace was 887 Hz

Addition of mannitol or choline chloride to the ACSF resulted in comparable decreases in oscillation frequency (Fig. 2). The similarity of the effects of mannitol and choline chloride suggests that chloride ions make a negligible contribution to *f*_Pn_. On the other hand, increases in [Na^+^]_out_ (independently of whether the addition of NaCl salt was compensated for by changes in osmolarity by reduction of choline chloride or not) led to marked increases in oscillation frequency.

These findings underscore the need to compensate for changes in osmolarity of the perfusate. Yet, they demonstrate that compensation cannot be adequately achieved through changes in the sodium concentration. Thus, in the following experiments, we compensated for changes in [K^+^]_out_ by equimolar changes in choline chloride.

### Frequency changes of Pn oscillations induced by alteration of the extracellular potassium concentration

**Fig. 3:**
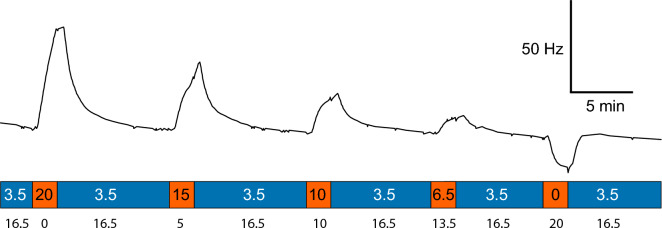
Time–frequency trace of the synchronized neural oscillations of the Pn in response to changes in [K^+^]_out_ (orange bands), relative to [K^+^]_out_ = 3.5 mM (blue bands). Concentrations (given in mM) of K^+^ were altered as indicated and applied for 2 min each. Black numbers below the color band denote concentration of choline chloride in mM used for osmolarity compensation. The oscillation frequency at the beginning of the trace was 817 Hz

To assess possible changes in the oscillation frequency induced by alteration of [K^+^]_out_, the Pn was perfused with ACSF containing 3.5 mM K^+^ (baseline [K^+^]_out_). Then, the nucleus was flushed for 2 min with ACSF containing either 0, 6, 10, 15, or 20 mM K^+^. The osmolarity of the six solutions was kept constant by addition of choline chloride (except in the case of [K^+^]_out_ = 20 mM). Changes in Pn oscillation frequency became evident within a few seconds after the flushing of the Pn with the altered K^+^ concentration started (Fig. 3). Such treatment resulted in a reduction of the frequency when the nucleus was exposed to [K^+^] = 0 mM, and in concentration-dependent increases in the frequency when the Pn was flushed with [K^+^] > 3.5 mM.

**Fig. 4:**
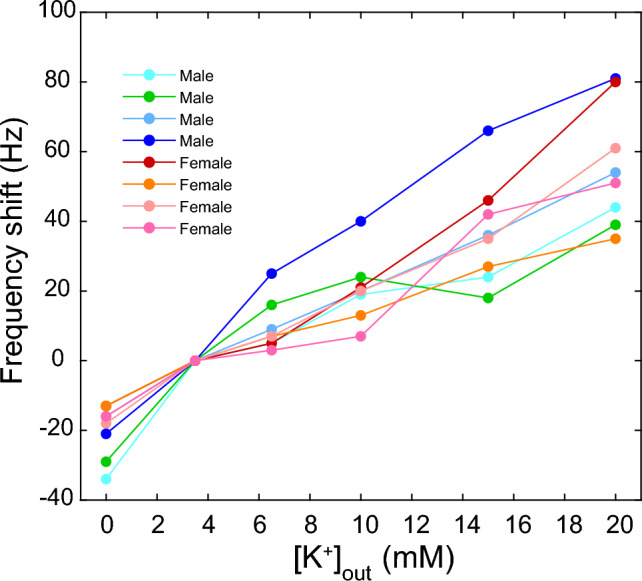
Effect of [K^+^]_out_ on the frequency of the Pn from 8 individuals. The isolated Pn was maintained in ACSF containing 3.5 mM K^+^. Then, the effect of 5 concentrations of K^+^ was assessed by flushing the Pn with the respective solution for 2 min, and by determining the induced maximum frequency shift, relative to the baseline frequency of the Pn immediately before the change in [K^+^]_out_. The frequency at [K^+^]_out_ = 3.5 mM (the concentration in the baseline perfusate) is set to a frequency shift of 0 Hz

To determine the frequency shifts induced by exposure of the Pn to the altered extracellular potassium concentration, we compared the baseline frequency immediately before the concentration change with the maximum frequency observed within 3 min after switching from the ACSF with [K^+^] = 3.5 mM to the ACSF with the altered [K^+^] (Fig. 4). In each of the 8 fish examined, the relative frequency change was significantly correlated with [K^+^]_out_ (Pearson correlation coefficients of 0.879–0.997; *p* < 0.05, two-tailed).

### Sex-dependent differences in Pn frequency shifts induced by changes in extracellular potassium concentration

Since the 8 fish used for analysis in this paper were unambiguously sexed as males (4 fish) and females (4 fish) based on their *f*_EOD_ values, and in 3 fish of each group the identification was additionally confirmed by gonadal inspection (cf. ‘Materials and methods—Animals,’ above), we examined whether sex-dependent differences existed in the relative degree of frequency shifts induced by alteration of [K^+^]_out_. To assess possible differences, we compared the frequency shifts of males and females after normalizing their values to those of the females. A reduction in [K^+^]_out_ from 3.5 mM to 0 mM resulted in a 32% larger frequency reduction in males compared to that in females. The frequency increases induced by increasing [K^+^]_out_ from 3.5 mM to 6 mM and 10 mM were 104% and 35%, respectively, larger in males than in females. Increases of [K^+^]_out_ to 15 mM and 20 mM produced virtually identical responses by the two sexes (the frequency decreases were 8% respective 3% lower in males than in females). The larger frequency shifts in males than females in response to smaller (≤ 10 mM) changes in [K^+^]_out_ are evident in Fig. 4, in which frequency responses before normalizations are plotted.

## Discussion

In the present study, we tested the hypothesis that changes in [K^+^]_out_ induce alterations in *f*_Pn_. This was achieved by using an in vitro preparation of the Pn and by assessing through extracellular recordings possible effects of changes in [K^+^]_out_ in the perfusate on this frequency, while maintaining the osmolarity of the medium.

### Effects of changes in osmolarity and [Na^+^]_out_ on Pn oscillation frequency

The initial experiments of the present study showed that an acute increase in osmolarity of the perfusate of the Pn preparation prompted a rapid decrease in oscillation frequency. This result is congruent with the well-established idea that the excitability of the brain is highly sensitive to acute alterations of the extracellular osmolarity (for reviews see Andrew 1991; Schwartzkroin et al. 1998). For example, in rat hippocampal slices reduction in extracellular osmolarity leads to hyperexcitability and enhanced epileptiform bursting, while this effect can be abolished by reversal of the hyposmolar state through mannitol (Andrew et al. 1989; Roper et al. 1992).

Our initial observations underscored the need to compensate for changes in osmolarity whenever [K^+^]_out_ is altered. However, such compensation cannot be adequately achieved by reduction of the concentration of NaCl in the perfusate, as was done by Dye (1991) because our initial experiments also demonstrated that changes in [Na^+^]_out_ have a pronounced impact on the oscillation frequency of the Pn. This effect was also seen when changes in NaCl concentration were compensated for by equivalent changes in osmolarity through addition or reduction of choline chloride. While these findings are in line with the notion that sodium currents are involved in the regulation of the pacemaker rhythm (Smith and Zakon 2000), they stress the need for compensation of osmolarity changes without alteration of [Na^+^]_out_. Thus, in the subsequent experiments during which we changed [K^+^]_out_, this was done by proper adjustment of the choline chloride concentration in the ACSF.

### Effects of changes in [K^+^]_out_ on Pn oscillation frequency

In the current study, we demonstrated that a 10 mM reduction in [K^+^]_out_ results in an approximately 25 Hz reduction in *f*_EOD_ over a [K^+^]_out_ range of 0 to 20 mM (Fig. 4), indicating that the K^+^-buffering hypothesis can explain at least partially the sexual dimorphic *f*_EOD_ in *A. leptorhynchus.* Yet, to achieve the marked average difference in *f*_EOD_ of approximately 140 Hz between males and females through modulation of buffering, it would be necessary that [K^+^]_out_ assume values with differences between the sexes that far exceed the normal variability within other vertebrate brain systems, which ranges from approximately 3.5–5 mM. The [K^+^]_out_-*f*_Pn_ relation is likely to be mediated by potassium ion channels in the pacemaking neurons and/or the associated astrocytic glia. Involvement of potassium ion channels in controlling *f*_Pn_ in *A. leptorhynchus* was demonstrated with potassium channel blockers (Smith and Zakon 2000). Identification of the specific types of potassium channels will be facilitated by the results of RNA-sequencing in the central nervous system of *A. leptorhynchus*, which revealed a total of 74 K^+^ channel genes (Salisbury et al. 2015).

We hypothesize that in our in vitro experiments the observed effects of [K^+^]_out_ on *f*_Pn_ were possible because (i) the perfusate reached major portions of the extracellular space and the capillaries of the submerged tissue preparation rapidly; (ii) the volume of the perfusate was so large, and its flow rate was so high, that buffering by astrocytes had a rather minor impact on the regulation of [K^+^]_out_. This notion is supported by two observations. First, *f*_Pn_, which was measured with the baseline perfusate containing 3.5 mM K^+^, was 93 ± 25 Hz (mean ± standard deviation; *n* = 8 fish) lower than f_EOD_ measured in intact fish. Second, the [K^+^]_out_-*f*_Pn_ function (Fig. 4) showed a linear relation over a wide range of [K^+^]_out_, including 0 mM.

### Sex-dependent differences in Pn frequency shifts induced by changes in extracellular potassium concentration

Analysis of the shifts in *f*_Pn_ induced by changes in [K^+^]_out_, relative to the arbitrarily chosen standard concentration of 3.5 mM, indicated some notable sex-dependent differences in the degree of these shifts at concentrations ≤ 10 mM. Concentration changes from 3.5 mM to 0 mM, 6 mM, and 10 mM resulted in *f*_Pn_ changes that were, on average, larger in males than in females. This finding is consistent with the previous observations that the association of astrocytes with the pacemaker and relay cells within the Pn is markedly higher in females than in males, and that this difference, presumably, results in better buffering of K^+^ ions in the extracellular space in females (Zupanc et al. 2014).

Although the relative differences between males and females in the *f*_Pn_ shifts induced by alteration of [K^+^]_out_ are pronounced, ranging from 32 to 104%, they translate to absolute values of less than 1 Hz in each of the 3 experiments. As mentioned in the previous section of the Discussion, in our experiments the buffering by the astrocytes of extracellular K^+^ ions is, likely, heavily obscured by the large volume of the perfusate and its high flow rate. We, therefore, interpret the small absolute differences in *f*_Pn_ shifts between males and females as a consequence of these experimental conditions. Nevertheless, the existence of sex-dependent differences under acute experimental conditions, and the sizeable degree of these differences in relative terms, are compatible with the notion that in situ similar differences in the buffering capacity of astrocytes translate into differences in [K^+^]_out_, thereby ultimately causing (at least partially) the sexual dimorphisms in *f*_Pn_, and *f*_EOD_.

### Perspectives

The main result of the present study—*f*_Pn_ correlates positively with [K^+^]_out_—is consistent with the hypothesis that differences between males and females in astrocyte-mediated buffering cause differences in [K^+^]_out_, which lead to shifts in *E*_K_, thereby ultimately establishing the sexual dimorphism in *f*_EOD_. For such a mechanism to generate the required male–female-related differential distribution of *f*_EOD_ within the species-specific range of 650–1,000 Hz, a substantial variability in [K^+^]_out_ among individual fish is required, from a few millimolar in females to several tens of millimolar in males.

Variations in [K^+^]_out_, correlating with different physiological states of an organism, have recently been found in murine models of Alzheimer’s disease, amyotrophic lateral sclerosis, and Huntington’s disease. In each of these disease models, [K^+^]_out_ was elevated, compared to healthy wild-type mice (Ding et al. 2024). Yet, these differences in [K^+^]_out_ were < 1 mM. Thus, to further test the hypothesis that [K^+^]_out_ mediates the sexual dimorphism in *f*_Pn_ (and thereby in *f*_EOD_), it will be imperative to determine [K^+^]_out_ locally in the Pn and to check whether this concentration differs between males and females.

Investigations in a few (non-electric) fish have shown that the range over which [K^+^]_out_ varies across different species is rather narrow (1.7–3.6 mM; Nilsson et al. 1993; Rice and Nicholson 1988). These values are reminiscent of the resting K^+^ concentration in the extracellular fluid of the mammalian brain, which is approximately 3 mM (Lux and Neher 1973; Prince et al. 1973; Moody et al. 1974). Nevertheless, given the continuously high-frequency (~ 1 kHz) oscillations of the Pn of *A. leptorhynchus* throughout the fish’s life, it appears well possible that [K^+^]_out_ in this nucleus is significantly higher than the concentrations known from the vast majority of other brain systems that fire only intermittently and/or at much lower frequencies. Experiments in leech have indicated that the generation of a single action potential results in an increase in [K^+^]_out_ of approximately 0.8 mM (Baylor and Nicholls 1969). Although this value is not directly transferable to explain the dynamics of [K^+^]_out_ fluctuations in the pacemaker nucleus, due to the many morphological and physiological differences between the two systems, it nevertheless provides a first indication of the large amount of K^+^ that is likely to exit from the firing pacemaking neurons into the extracellular space. This amount of K^+^ in the extracellular space plays a critical role by providing the substrate for alteration through modulation of the buffering capacity of astrocytes. Such a mechanism would be well suited for regulation of the extracellular potassium dynamics, and thereby for the establishment of the sexual dimorphism in the distribution of the *f*_EOD_ of individual fish. However, further experiments, including direct measurement of [K^+^]_out_ in the Pn of males and females, and experimental modulation of the K^+^ buffering capacity of the astrocytic syncytium in this nucleus, will be necessary for a definite conclusion on this point.

## Data Availability

Data that support the findings of this study are available from the corresponding author upon request.

## Abbreviations

ACSF: Artificial cerebrospinal fluid
EOD: Electric organ discharge
*f*_EOD_: Frequency of the electric organ discharge
*f*_Pn_: Frequency of the synchronized neural oscillations of the pacemaker nucleus
GFAP: Glial fibrillary acidic protein
K^+^: Potassium ions
[K^+^]_out_: Extracellular potassium concentration
[Na^+^]_out_: Extracellular sodium concentration
Pn: Pacemaker nucleus
